# Effect of COVID-19 on Health-Related Quality of Life in Adolescents and Children: A Systematic Review

**DOI:** 10.3390/ijerph18094563

**Published:** 2021-04-25

**Authors:** Hadi Nobari, Mohamad Fashi, Arezoo Eskandari, Santos Villafaina, Álvaro Murillo-Garcia, Jorge Pérez-Gómez

**Affiliations:** 1 Department of Physical Education and Sports, University of Granada, 18010 Granada, Spain; 2 Department of Exercise Physiology, Faculty of Sport Sciences, University of Isfahan, Isfahan 81746-7344, Iran; 3 HEME Research Group, Faculty of Sport Sciences, University of Extremadura, 10003 Cáceres, Spain; jorgepg100@gmail.com; 4 Department of Biological Sciences in Sports, Faculty of Sport Sciences and Health, Shahid Beheshti University, Tehran 198396-3113, Iran; 5 Department of Exercise Physiology, Faculty of Physical Education and Sports Science, Tehran University, Tehran 1417935840, Iran; A.eskandari_1988@yahoo.com; 6 Physical Activity and Quality of Life Research Group (AFYCAV), Faculty of Sport Sciences, University of Extremadura, Av. De Universidad s/n, 10003 Caceres, Spain; svillafaina@unex.es (S.V.); alvaromurillo@unex.es (Á.M.-G.)

**Keywords:** adolescent, COVID-19, children, HRQoL, lifestyle, wellbeing

## Abstract

The aim of the present systematic review was to assess and provide an up-to-date analysis of the impact of coronavirus disease 2019 (COVID-19) pandemic on the health-related quality of life (HRQoL) of children and adolescents. Thus, an electronic search of the literature, in two well-known databases (PubMed and Web of Science), was performed until February 2021 (without date restriction). PRISMA guideline methodology was employed and data regarding the HRQoL were extracted from eligible studies. Articles were included if they met the following inclusion criteria: (a) children and/or adolescent population (4 to 19 years old); (b) HRQoL as a main assessment; (c) German, Spanish, Portuguese, French, and English language; and (d) pre-pandemic and during pandemic HRQoL data. Following the initial search, 241 possible related articles were identified. A total of 79 articles were identified as duplicates. Moreover, 129 articles were removed after reading the title and abstract. Of the remaining 33 articles, 27 were removed since they were not focused on children or adolescents (*n* = 19), articles did not report pre- and post- pandemic HRQoL values (*n* = 6), articles were not focused on HRQoL (*n* = 6), and one article was an editorial. Finally, six studies fulfilled the inclusion criteria and, therefore, were included in the systematic review. A total of 3177 children and/or adolescents during COVID-19 were included in this systematic review. Three articles showed that COVID-19 pandemic significantly impacted the HRQoL of children and adolescents, and another did not report comparison between pre- and during COVID-19 pandemic, although a reduction in the HRQoL can be observed. Nevertheless, two articles did not find significant changes and another one did not report *p*-values. Regarding sex differences, only two studies analyzed this topic, observing no differences between girls and boys in the impact of COVID-19 pandemic on HRQoL. Taking into account these results, this systematic review might confirm that COVID-19 has a negative impact on the HRQoL of children and/or adolescents.

## 1. Introduction

Health-related quality of life (HRQoL) has been defined as the level of wellbeing consequent from the evaluation that a person makes of diverse domains of his life, considering the impact these have on his health status. It is characterized as subjective, multidimensional, and changing over time [1]. The HRQoL assessment incorporates at early ages the perception of physical, psychological, and social wellbeing according to evolutionary development and individual differences, within a specific cultural context, and considers the ability to fully participate in the activities and the physical, social, and psychosocial functions appropriate to their age. Children with poor HRQoL are less likely to develop normally and mature into healthy adults [2].

Many children and adolescents in developed countries lead sedentary lifestyles, reduced active leisure activities, and increased reliance on sedentary lifestyles [3]. Before the COVID-19 pandemic, a previous study stated that 81% of students aged 11–17 years were insufficiently physically active [4]. Independently of physical activity levels, sedentary activities, especially those based on the use of electronic devices, are associated with an increased risk of obesity, a reduction in physical condition, self-esteem, and prosocial behavior [5]. In this regard, the COVID-19 pandemic has increased the amount of screen recreational time [6,7]. This is quite relevant since a previous systematic review of reviews indicated that the evidence that screen time was associated with poorer HRQoL was moderate [8].

In addition to inactivity, stressful situations such as coronavirus disease 2019 (COVID-19) pandemic have affected the HRQoL of children. Home quarantine conditions and the use of the Internet or mobile phones have also been effective in creating these conditions. The COVID-19 pandemic has led to rapid, unprecedented changes to the lives of billions of children and adolescents [9,10].

The impact of pandemic COVID-19 on HRQoL is not yet understood sufficiently well. Children and adolescents face massive changes in their daily lives, including school closures, home confinement, and social distancing rules, which can burden them substantially [11,12]. Managing with the current situation and complying with the current restrictions on top of this can be especially difficult for children and adolescents since these circumstances can be experienced as being incongruent with their developmental tasks. The challenges and consequences of COVID-19 might therefore have a tremendous impact on their HRQoL [13]. In this regard, previous to COVID-19 pandemic, anxiety was the main mental disorder in both, children, and adolescents, with more than 15% of this population affected by this disorder [14].

Furthermore, as commented before, restriction in mobility, social distancing, or school closures have been increased sedentary behavior. Thus, it is expected that HRQoL would be reduced in children and adolescents. Previous studies have indicated that HRQoL have been reduced due to COVID-19 [15,16] whereas others did not find differences [17,18]. However, to the best of our knowledge, no systematic review has synthesized the existing results in order to clarify this divergence. To aim of the present systematic review was to assess and provide and up-to-date analysis of the impact of the coronavirus disease 2019 (COVID-19) pandemic on the health-related quality of life (HRQoL) of children and adolescents.

## 2. Materials and Methods

The present systematic review was conducted following the guidelines included in the Preferred Reporting Items for Systematic Reviews and Meta-Analyses Guidelines (PRISMA), for search procedures, study selection, and data collection and analysis [19]. To achieve the objective of this research we systematically reviewed the literature to analyze observational studies which compared the HRQoL data of the same children and adolescents pre and post/during COVID-19 pandemic. This would allow us to analyze if COVID-19 pandemic had a negative effect on HRQoL in children and adolescents.

### 2.1. Data Sources and Searches

Two well-known databases, PubMed and Web of Science (where Current contents connect, Derwent innovations index, Korean journal database, Medline, Russian science citation index, and SciELO citation index are included), were used to identify all the observational studies, published until 8 February 2021, evaluating HRQoL during COVID-19 pandemic in children and/or adolescents. The search string used in all databases was (“coronavirus”[MeSH Terms] OR “coronavirus”[All Fields] OR “coronaviruses”[All Fields] OR (“sars cov 2”[MeSH Terms] OR “sars cov 2”[All Fields] OR “covid”[All Fields] OR “covid 19”[MeSH Terms] OR “covid 19”[All Fields])) AND “Quality of life”[All Fields] AND (“child”[MeSH Terms] OR “child”[All Fields] OR “children”[All Fields] OR “child s”[All Fields] OR “children s”[All Fields] OR “childrens”[All Fields] OR “childs”[All Fields] OR (“adolescences”[All Fields] OR “adolescency”[All Fields] OR “adolescent”[MeSH Terms] OR “adolescent”[All Fields] OR “adolescence”[All Fields] OR “adolescents”[All Fields] OR “adolescent s”[All Fields])).

Three researchers (M.F., A.E., and H.N) independently conducted the search. Discrepancies were solved by a discussion with a fourth investigator (A.D).

**Figure 1:**
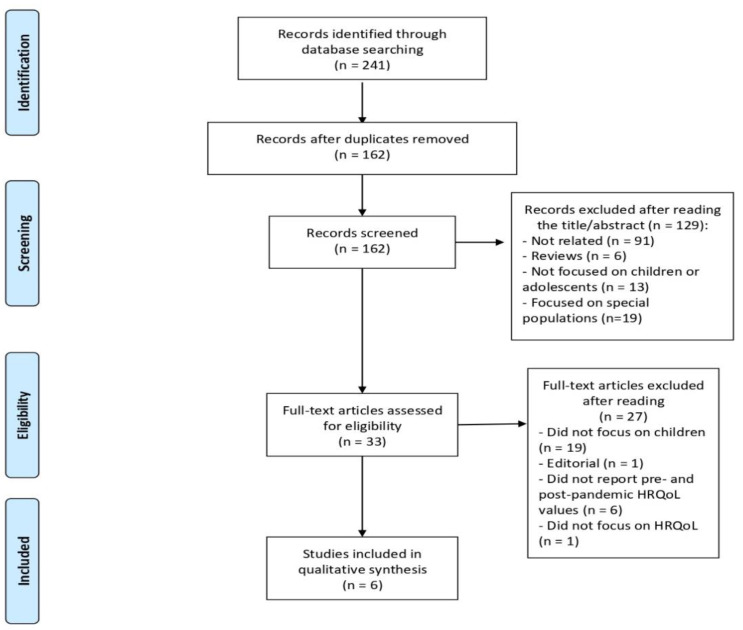
Flow chart of the selection process in this systematic review.

Figure 1 shows the article selection process. The studies were included if they met the following inclusion criteria: (a) children (0–10 years-old) and/or adolescents (10–19 years-old) population [20]; (b) HRQoL as a main assessment; (c) German, Spanish, Portuguese, French, and English language; and (d) pre-pandemic and during pandemic HRQoL data. Exclusion criteria were (a) abstracts, editorials, comments, reviews, and guidelines, and (b) participants were suffering from a major health condition such as cancer or disabilities.

### 2.2. Risk of Bias

The Evidence Project risk of bias tool [21] has been used to assess the risk of bias. This scale consisted of eight items, assessed as no, yes, not applicable, or not reported. The eight items are: (1) cohort; (2) control or comparison group; (3) pre-post intervention data; (4) random assignment of participants to the intervention; (5) random selection of participants for assessment; (6) follow-up rate of 80% or more; (7) comparison groups equivalent on sociodemographic; and (8) comparison groups equivalent at baseline on outcome measures. Two authors (A.M. and A.D.) evaluated the risk of bias independently, and disagreements were solved by a discussion with another author (J.P.-G.).

### 2.3. Data Extraction

We developed a data extraction sheet (based on the study design, sample size, country, population—children or adolescents, instrument for assessing HRQoL, pre and during HRQoL values). One review author (S.V) extracted the following data from included studies and a second author checked the extracted data (A.M.-G). Disagreements were resolved by a discussion with another author (J.P.-G.).

### 2.4. Evidence Synthesis and Data Analysis

Synthesis of results are presented with an initial description of the study population (children or adolescents), including total sample size and age range, as well as the study design of included studies. Then, instruments used to assess the HRQoL and a general summary of COVID-19 impact on HRQoL of children and adolescents included in each study will be presented. After that, for each study a simple summary with main results will be extracted. Due to the study design and heterogeneity of included articles, an estimated effect across studies with a confidence interval could not be calculated.

## 3. Results

### 3.1. Study Selection

Figure 1 reports the PRISMA flow diagrams of the study selection. Following the initial search, 241 possible related articles were identified. A total of 79 articles were identified as duplicates. Moreover, 129 articles were removed after reading the title and abstract (see Figure 1 for reasons). Of the remaining 33 articles, 27 were removed since they were not focused on children or adolescents (*n* = 19), articles did not report pre- and post- pandemic HRQoL values (*n* = 6), articles were not focused on HRQoL (*n* = 6), and one article was an editorial. Finally, six articles were included in the systematic review (see Figure 1). Details and characteristics of these articles are also provided in Table 1.

### 3.2. Risk of Bias

The Evidence Project risk of bias tool was employed in the present study (see Table 1). This tool evaluated the risk of bias of the six articles included in this systematic review. After evaluating all of them, six articles fulfilled criteria one and three (cohort and pre-post measurements). Three articles fulfilled the criteria related to the follow-up rate of 80% or more. However, the most critical concerns are related to the random assignment of participants to the intervention and the random selection of participants for assessment. Two criteria, the comparison group’s equivalent on sociodemographic measures, and the comparison groups equivalent at baseline on outcome measures, were not assessed due to the observational design of the studies included in this systematic review.

### 3.3. Synthesis of Results

Table 2 summarizes the impact of the COVID-19 pandemic on the HRQoL of children and adolescents. A total of 3177 children and adolescents have been included in this systematic review. The age ranged between 4 to 18 years.

Regarding the instruments used to evaluate the HRQoL, four different scales were used to evaluate the HRQoL of children and adolescents: KIDSCREEN-10 index, the SF-36, the Pediatric Quality of Life Inventory Questionnaire, and a Likert Scale (0–10). Three articles explicitly stated that used language validated versions [16,22] whereas the other three articles only described the questionnaire in their article [15,17,18]. To summarize, three articles reported that COVID-19 significantly reduced the HRQoL of children and adolescents [15,16], two articles did not find a significant impact [17,18], and one article did not report statistical comparison between pre- and post-pandemic data. Differences between girls and boys were explored in two articles [16,22] and non-significant differences were observed in the effect of the COVID-19 pandemic on the HRQoL. The impact of COVID-19 on the different dimensions of HRQoL was only analyzed in one study [16], showing that COVID-19 has negatively affected functional capacity, physical aspect, general health status, vitality, social aspects, emotional aspects, and mental health in both children and adolescents. However, neither of the studies analyzed whether the COVID-19 pandemic has equally impacted the HRQoL of children and adolescents.

The KIDSCREEN-10 index covers the physical, social, and psychological spheres of health, providing a global HRQoL score [23]. Its ten items (e.g., “Have you felt full of energy?”) were presented with 5-point response scales (0 = “never” to 4 = “always” or 0 = “not at all” to 4 = “extremely”), having a mean score ranging from 0 to 4. The SF-36 measures the participants’ self-reported opinion about their physical and mental well-being [24]. It has eight domains of HRQoL: general health, energy/vitality, body pain, physical functioning, emotional well-being, role limitations due to physical health, role limitations due to emotional problems, and social functioning. Responses to each question within a domain are combined to generate a score from 0 to 100, where 100 indicates “good health”. The 23-item PedsQL™ 4.0 Generic Core Scales [25] contain four dimensions with different items: (1) Physical Functioning (8 items), (2) Emotional Functioning (5 items), (3) Social Functioning (5 items), and (4) School Functioning (5 items). A five-point response scale is in the parent proxy-report (0 = never a problem; 1 = almost never a problem; 2 = sometimes a problem; 3 = often a problem; 4 = almost always a problem). Items are reverse-scored and linearly transformed to a 0–100 scale. The Likert scales, with a 0–10 scale, which was used to measure the HRQoL, represented the 0 as the absence of health and 10 representing complete health. In this systematic review, three articles employed the KIDSCREEN-10 index to assess the quality of life of children and adolescents [13,18,22], one article used the SF-36 [16], another the Pediatric Quality of Life Inventory Questionnaire [17], and another used a Likert Scale (0–10) [15].

Three articles reported that COVID-19 significantly reduced the HRQoL of children and adolescents [15,16]. Dragun, Veček, Marendić, Pribisalić, Đivić, Cena, Polašek and Kolčić [15] analyzed a total of 531 adolescents. Their results showed that lockdown significantly affected HRQoL, happiness, optimism (*p* < 0.001), as well as perceived stress. Interestingly, the Mediterranean diet adherence positively correlated to HRQoL pre and during COVID-19 lockdown. Therefore, this study concludes that given the numerous beneficial effects associated with the Mediterranean diet adherence, modification of lifestyle through application of lifestyle medicine deserves a priority approach. In the study of de Matos, Aidar, Almeida-Neto, Moreira, Souza, Marçal, Marcucci-Barbosa, Martins Júnior, Lobo and dos Santos [16], a total of 69 children and adolescents participated in an online survey, based on the SF-36, in order to evaluate the HRQoL. Results showed that HRQoL significantly decreased in all aspects of quality of life analyzed by the SF-36 in both adolescents and children. Authors concluded that the scenario of social isolation negatively impacted the level of physical activity, quality of life, and stress level in Brazilians. Thus, implementing an adapted physical training program at home during the period of the pandemic may help to decrease the negative physiological and psychological impact of inactive behaviors. Ravens-Sieberer, Kaman, Erhart, Devine, Schlack and Otto [13] analyzed a total of 793 children and adolescents using the KIDSCREEN-10 questionnaire. This study showed detailed data regarding the reduction of HRQoL due to the COVID-19 pandemic. In this regard, authors pointed that before the pandemic, 15.3% (*n* = 146; based on weighted data of the BELLA study) of children and adolescents reported low HRQoL whereas, during the pandemic, 40.2% of the children and adolescents reported low HRQoL (*n* = 418; based on weighted self-reported data of the COPSY study). Furthermore, a gender stratified analysis showed that a higher proportion of girls reported low HRQoL, when compared to their male peers both before and during the pandemic. Interestingly, younger children were affected significantly more than older ones. For instance, the percentage of children reporting low HRQoL ranged from 7.7% to 41.3% in 11- to 13-year-old children and from 17.1% to 39.3% in 14- to 17-year-olds. Taking into account all these results, authors concluded that health promotion and prevention strategies are needed to maintain children’s and adolescents’ mental health, as well as to improve their HRQoL, and mitigate the burden caused by COVID-19, particularly for children who are most at risk.

Two articles did not find a significant impact [17,18] of COVID-19 pandemic on HRQoL. A total of 40 children and adolescents participated in the study of Abawi, Welling, van den Eynde, van Rossum, Halberstadt, van den Akker and van der Voorn [17]. The 23-item Paediatric Quality of Life inventory (PedsQL) 4.0 (parents’ proxy-report version) was registered. The mean PedsQL total score between baseline and COVID-19 outbreak decreased in children, although the change was not statistically significant (mean change −6.3 ± 29.9; *p* = 0.26). Nevertheless, authors also study anxiety and showed a bigger decrease in children for whom anxiety was reported vs. those who did not (mean change −10.3 ± 36.5 vs. −3.3 ± 24.4). However, it was also not statistically significant. Thus, authors concluded that healthcare professionals should address possible COVID-19 related anxiety in children. Moreover, in the study of Vallejo-Slocker, Fresneda and Vallejo [18], a total of 33 children and adolescents completed the KIDSCREEN-10 questionnaire in order to assess the HRQoL. Results showed that no significant differences were detected between pre- and during COVID-19 pandemic in the HRQoL. Interestingly, authors analyzed the possible differences between sexes. In this regard, girls scored higher (*n* = 223, M = 3.8; SD = 2.46) than males (*n* = 236, M = 2.9, SD = 2.17) in emotional problems, indicating worse functioning. In the same line, using the KIDSCREEN-10 total index, boys obtained higher results (*n* = 236, M = 51.04; SD = 9.89) than girls (*n* = 223, M = 48.89; SD = 10.01), indicating higher HRQoL. Thus, authors concluded that it is necessary to monitor the mental health status of children and adolescents in order to prevent possible problems.

Lastly, in the study of Wunsch, Nigg, Niessner, Schmidt, Oriwol, Hanssen-Doose, Burchartz, Eichsteller, Kolb and Worth [22], a total of 1711 children and adolescents were screened using the KIDSCREEN-10 instrument to assess their HRQoL. In this study, authors did not present a t-test to compare pre and during HRQoL, but reported whether physical activity, screen time, and HRQoL before COVID-19 predicted physical activity, screen time, and HRQoL during the COVID-19 pandemic. This analysis revealed that physical activity during-COVID-19 was positively predicted by pre-COVID-19 HRQoL (standardized estimate = 0.07; *p* = 0.003) and negatively predicted by pre-COVID-19 screen time (standardized estimate = −0.21; *p* < 0.001). Moreover, when the information were stratified by gender, screen time pre- COVID-19 negatively predicted during-COVID-19 physical activity (standardized estimate = −0.24; *p* < 0.001), but no statistically significant relationship between HRQoL and physical activity was observed (*p* = 0.112). For females, physical activity during-COVID-19 was positively predicted by HRQoL pre-COVID-19 (standardized estimate = 0.09, *p* = 0.007) and negatively by leisure screen time pre-COVID-19 (standardized estimate = −0.18, *p* < 0.001). Nevertheless, authors compared their results with European norms and German children, observing a decrease. For instance, prior to the COVID-19 lockdown, mean T-Scores were 44 points at the 27th percentile, and during the lockdown, scores decreased to 41 points, reflecting the 18th percentile. Therefore, authors concluded that policies to improve the HRQoL, especially in children aged 4 to 10 years and females, should be promoted in order to increase resilience. Moreover, due to the negative association between pre-COVID-19 screen time and within-COVID-19 physical activity, health policy must implement counter measures to reduce screen time in children and adolescents. 

## 4. Discussion

The purpose of this systematic review was to provide an up-to-date analysis of the impact of COVID-19 pandemic on the HRQoL of children and adolescents. Thus, articles that analyzed pre and post/during pandemic HRQoL values were included. Findings suggest that children and/or adolescents experienced a reduction in the HRQoL due to the COVID-19 pandemic. 

The COVID-19 pandemic, due to the high uncertainty and stress, has decreased psychological health [26]. Previous studies have found a significant impact of COVID-19 confinement on stress, depression, or anxiety [27,28]. This decrement in psychological health has also been observed in children and adolescents [29,30]. Furthermore, children and adolescents experienced massive changes in their daily lives, such as school closures, social distancing, or home confinement, significantly decreasing the HRQoL [12,31]. In this regard, previous studies have indicated that HRQoL has been reduced due to the COVID-19 pandemic [16,32,33]. The results of this systematic review are in the same line, with three articles showing a significant reduction of HRQoL in children and adolescents. Moreover, two studies independently explored the impact of COVID-19 in girls and boys [13,22]. Results showed that indifferently to their sex, HRQoL was reduced. However, a previous study showed that girls were more likely to experience decreased mental health than boys during COVID-19 pandemic [29]. In the same line, previous studies showed that girls are more vulnerable to psychological distress than boys [34]. Therefore, future studies should explore sex-based differences on the impact of the COVID-19 pandemic in children and adolescents.

Notably, due to the governments’ social isolation and the measures to control the spread of COVID-19, previous studies have detected a significant decrease in physical activity level [35,36]. These measures have limited the opportunity to stay physically active. Due to this fact, Costa, et al. [37] showed that during the pandemic, Brazilians’ level of physical activity was significantly reduced, becoming harmful to the health of the population [37]. Sustained physical inactivity is usually associated with decreased physical and mental wellbeing and elevated disease-specific behavior and all-cause mortality risk [38].

As commented before, social isolation can lead to a reduction in social contact and longer periods of immobility. This would lead, directly or indirectly, to increase the use of interactive devices, such as TV, computers, and mobile phones [39]. Previous studies have found that the total amount of recreational screen time has increased during the COVID-19 pandemic [6,7]. In this regard, a previous study showed that children and adolescents increased a total of 61.2 minutes per day of recreational screen time due to the COVID-19 pandemic [40]. This study also found that boys spend more recreational screen time per day than girls, 66.2% and 56.3%, respectively. Interestingly, this article showed that favorite recreational screen activity is TV whereas, for adolescents, the Internet’s use was predominately chosen. This is quite relevant, especially in adolescents where sexting (exchange of a message containing sexual content via internet) has been shown to be prevalent among juveniles [41]. Increased time on recreational internet could have increased this behavior with negative consequences on children’s mental health and HRQoL. 

Social isolation can enhance loneliness and abandonment, triggering adverse behavioral relationships (i.e., aggressiveness, crying, emotional pain, etc.) [42]. Moreover, the absence of social interaction can affect the emotional perception that will influence the feeling of vitality and the perception of general health. This has a direct influence on mental health, and all these interconnected reactions can be harmful to the physical aspects, which can reduce the functionality and increase the perception of physical pain [43]. Therefore, the self-perceived quality of life tends to suffer negative changes during the isolation period of the COVID-19 pandemic [44]. Sieberer et al. showed that lower HRQoL and more mental health problems during pandemic COVID-19 and health promotion and prevention strategies need to be executed [13].

Previous studies have explored the benefits of physical exercise to combat the negative impact of the COVID-19 pandemic [45]. In this regard, a previous study found that those who were not enrolled in physical exercise during COVID-19 confinement showed a higher level of stress, anxiety, and depression [9]. In the same line, a previous randomized controlled trial which focused on the effects of two home-based physical exercise interventions (using high-intensity interval training and moderate-intensity training) showed that both groups reduced stress, anxiety, and depression as well as increased resilience. However, the group who performed high-intensity interval training significantly obtained the greatest reduction of depression when compared to moderate-intensity training. Regarding resilience, the significant effect on resilience is quite important to face the stressful and uncertain situation caused by the COVID-19 pandemic [46,47]. According to a systematic review, children should perform 60–180 min of daily moderate to vigorous physical activity and use their own bodyweight or light weights during strength training [48]. However, in children and adolescents who were not involved previous to the COVID-19 pandemic, it is recommended to continue moderate-intensity exercise every day for 10 to 15 min [49].

Furthermore, another relevant factor which should be considered in the decrease of HRQoL in children and adolescents are parents. In this regard, a previous study has shown how violence against children has increased under home confinement leaving children at risk of abuse and trauma [50], particularly in those families with a low socioeconomic status [51]. Furthermore, poorer families are less financially resilient and are more exposed to job and earnings losses while their children are likely to be disproportionally disadvantaged by school-closures [52]. Moreover, growing up in poorer neighborhoods increases the risk of catching the virus [52]. Additionally, the COVID-19 outbreak has increased the likelihood of financial and social insecurity for low-income groups, which could hypothetically contribute to low HRQoL of children and adolescents [52]. Thus, a previous study showed that parental job and income losses are associated with parents’ depressive symptoms, stress, diminished sense of hope, and negative interactions with children [53,54]. 

The principal limitation of this review is the small number of articles about HRQoL that compare pre and during pandemic values. In addition, the articles analyzed are from children and adolescents from different countries. This can be a limitation because each country has had different time and confinement restrictions. This consideration may be a future line of research when more studies appear on this topic. Additionally, articles in languages other than English, Spanish, German, French, or Portuguese were not included in this systematic review. Thus, it is plausible to suppose that some important pediatric cohorts were not included in our systematic review. Furthermore, one article included a Likert scale [15], while another included the SF-36 questionnaire to assess the HRQoL. In order to measure HRQoL in children and adolescents, other questionnaires such as PedsQL and KIDSCREEN-10 can be useful. Future studies should explore if COVID-19 has equally affected girls and boys, as well as adolescents and children. Additionally, studies should explore, in a deeper way, which aspects of the HRQoL have been more affected by the COVID-19 pandemic in children and adolescents.

Despite the limitation, this study has some strengths which should be acknowledged. First, this is the first systematic review which analyzes the impact of COVID-19 pandemic on the HRQoL of children and adolescents. Additionally, more than 3100 children and adolescents were included in this study. Lastly, this study showed how COVID-19 has negatively impaired the HRQoL of children and adolescents, so interventions are encouraged to recover the HRQoL level pre-pandemic.

## 5. Conclusions

The COVID-19 pandemic could have significantly decreased the HRQoL of children and adolescents. Regarding sex differences and HRQoL dimensions, strong evidence was not shown, so future studies should explore whether the COVID-19 pandemic equally impacted boys and girls as well as the most affected HRQoL aspect. Furthermore, interventions such as physical activity should be promoted in children and adolescents in order to recover the pre-pandemic HRQoL levels. 

## Figures and Tables

**Table 1:** Risk of bias of included articles using the Evidence Project risk of bias tool.

Study	1	2	3	4	5	6	7	8
Abawi (2020)	Yes	No	Yes	No	No	No	NA	NA
Dragun (2020)	Yes	No	Yes	No	No	No	NA	NA
Matos (2020)	Yes	No	Yes	No	No	Yes	NA	NA
Ravens-Sieberer (2021)	Yes	No	Yes	No	No	Yes	NA	NA
Vallejo-Slocker (2020)	Yes	No	Yes	No	No	No	NA	NA
Syzmanski (2018)	Yes	No	Yes	No	No	Yes	NA	NA

The eight items are (1) cohort; (2) control or comparison group; (3) pre-post intervention data; (4) random assignment of participants to the intervention; (5) random selection of participants for assessment; (6) follow-up rate of 80% or more; (7) comparison groups equivalent on sociodemographic; and (8) comparison groups equivalent at baseline on outcome measures. NA: non applicable.

**Table 2:** Main findings of children and/or adolescents HRQoL pre- and during COVID-19 pandemic.

Author	Country	Study Design	Sample Size (Girls)	Age (Years)	Questionnaire	HRQoL		*p*-Value
						Pre-Pandemic	During-Pandemic	
Abawi 2020	Netherlands	Cross-Sectional	40	10.5 (7.6–15.2)	PedsQL	66.2 (17.7)	Change of −6.3 (29.9)	0.26
Dragun (2020)	Croatia	Cross-Sectional	531 (384)	18 (6.0)	Likert scale	8.0 (2.0)	7.0 (2.0)	<0.001
Matos (2020)	Brazil	Cross-Sectional	25 children (10) 44 adolescents (25)	9.56 (1.47) 16.3 (1.67)	SF-36	-	-	<0.001 ^a^
Ravens-Sieberer (2021)	Germany	Cross-Sectional	793 (396)	12.25 (3.30)	KIDSCREEN-10	Low HRQol in Boys: 10.4%	Low HRQol in Boys: 35.7 %	<0.001
						Low HRQol in Girls: 20.4%	Low HRQol in Girls: 44.7 %	<0.001
						Low HRQol in Boys and girls: 15.3%	Low HRQol in Boys and girls: 40.2 %	<0.001
						Proxy results of 7-to 10-years-olds: 7.4%	Proxy results of 7-to 10-years-olds: 26.8%	<0.001
						Proxy results of 11-to 13-years-olds: 12.8%	Proxy results of 11-to 13-years-olds: 14.5%	<0.001
Vallejo-Slocker (2020)	Spain	Cross-Sectional	33	8–18	KIDSCREEN-10	50 (10)	50.4 (10.1)	0.4
Wunsch (2021)	Germany	Cross-Sectional	1711	4–10 11–17	KIDSCREEN-10	Male children 44.89 (3.92)	Male children 40.68 (4.33)	
						Female children 45.49 (4.88)	Female children 41.27 (4.34)	
						Male adolescents 43.87 (4.19)	Male adolescents 40.77 (4.70)	
						Female adolescents 43.15 (4.32)	Female adolescents 40.83 (4.27)	

^a^: There was a significant decrease in all aspects of quality of life analyzed by the SF-36 (Functional capacity, Limitation by physical aspect, General Health Status, Vitality, Social Aspects, Emotional Aspects, and Mental Health) in both children and adolescents. Data can be checked in the original article. HRQoL: health-related quality of life. COVID-19: coronavirus disease 2019. PedsQL: Pediatric Quality of Life Inventory Questionnaire.

## Data Availability

The datasets used and/or analyzed during the current study are available from the corresponding author on reasonable request.
